# A health financing policy agenda for climate mitigation and adaptation

**DOI:** 10.2471/BLT.23.291253

**Published:** 2024-05-01

**Authors:** Maarten Oranje, Inke Mathauer

**Affiliations:** a Amsterdam, Kingdom of the Netherlands.; b Department of Health Financing and Economics, World Health Organization, Avenue Appia 20, 1211 Geneva 27, Switzerland.

The international community increasingly acknowledges the contribution of health systems to climate change and environmental degradation: globally, the health sector contributed approximately 5% to greenhouse gas emissions in 2019.^1^ This contribution is mainly due to energy-intensive production and transportation of supplies such as drugs, commodities and equipment, as well as energy consumption within health facilities and waste treatment.^2^ Conversely, climate change, pollution and related consequences, including psychological stress, also affect human health in numerous ways. Among others, higher temperatures and changes in rainfall patterns lead to prolonged droughts or floodings as well as higher transmission rates of vector-borne and waterborne diseases, increasing health-care needs and often leading to increased out-of-pocket health expenditure.^3^ The World Health Organization (WHO) estimates that by 2030, the additional direct costs for the health sector alone will be 2–4 billion United States dollars (US$) per year globally.^2^

Climate change also has implications for health financing. The way health financing policies are designed can contribute to mitigation. Mitigation measures are actions that aim to reduce greenhouse gas emissions – thus tackling the main cause of climate change. At the same time, various health financing arrangements need to be adapted to address the rising health needs and expenditure. Some of these adaptations also support the necessary changes to make the health system resilient. Adaptation measures serve to adjust to actual or expected climate change and its effects, and to reduce vulnerability.

Sparse documentation is available on how climate financing is (or can be) used for the health sector.^4^ The recent WHO *Operational framework for building climate resilient and low carbon health systems*^2^ covers financing for climate change and health interventions. Here, we link to this WHO work and delve further into the health financing functions, that is, revenue raising, pooling and purchasing (Box 1). This paper is geared towards health financing policy-makers. To contribute to the conceptualization, awareness raising and agenda setting of the role of health financing, this article provides an initial overview of policy options for mitigation and adaptation measures within health financing that national or subnational governments could and/or already apply, with a focus on low- and middle-income countries. Finally, we identify some key questions that can help advance the policy and research agenda for health financing to support climate-resilient and low-carbon health systems. We emphasize that such health financing policy measures are only one part of the overall government and societal response, and need to be closely aligned with wider government action.

Box 1 **Definitions of revenue raising, pooling and purchasing**

- Revenue raising is the process of raising money to pay health system costs, for example through taxation and health insurance contributions.
- Pooling is the accumulation and aggregation of prepaid funds, so that the financial risk of having to pay for health care is shared by all members of the pool.
- Purchasing is the process of allocating these prepaid and pooled funds from purchasers to health-care providers through payment methods.^5^

## Policy options for health financing

### Revenue raising

#### Mitigation measures

While primarily the responsibility of the finance ministry, thus not a health financing policy per se, governments can tax goods and services with negative health or environmental effects (such as a carbon tax) or remove subsidies for fossil resources.^6^^–^^8^ Doing so would also affect the behaviour of both private sector actors and households, thereby contributing to climate change mitigation. These revenues could be used to finance climate mitigation and adaptation measures in health and other sectors. Governments can also diminish their dependence on oil and gas revenues that they collect through taxation and/or from state-owned companies, and shift to other sources for government health financing.^9^

#### Adaptation measures

Governments need to increase overall revenues for the health sector due to an increased burden of disease caused by climate change; they may also access existing climate funding that globally seems not yet sufficiently allocated to the health sector.^4^^,^^8^ Notably, many low- and middle-income countries will need to receive external funding to cover the costs of this increased burden in the short term. Additional public funding will also be needed for common goods of health such as surveillance, health emergency and climate disaster preparedness measures.

Climate change and disasters may force people to leave their homes and workplaces. To provide accessible and mobile health services, relying predominantly on general government revenues and a highly progressive tax system will be even more critical than in normal circumstances. Health insurance contributions would constitute an additional burden, especially for people in the informal economy and internally displaced people. 

### Pooling

#### Mitigation measures

Reducing fragmentation (that is, decreasing the differences in people’s health risks across different pools) and merging of health coverage schemes contribute to larger risk pools and thus to higher redistributive capacity, larger population coverage, reduced administrative costs and increased system efficiencies. These effects may also contribute to mitigation through lower resource consumption.

#### Adaptation measures

Better pooling and reducing fragmentation in the health financing system will be even more relevant in the future, to cope with the increased pressure on the health sector. Governments must be able to quickly adjust and modify the distribution of health funds across the country to meet the evolving health needs due to climate change. In addition to supplementary and contingency funds in case of emergencies, eligibility and targeting criteria as well as mechanisms and formulas for risk equalization or risk adjustment – that is, adjusting pool funding according to health needs and risks – should be adaptable during adverse climate events and related health emergencies,^10^ to consider people most affected and at higher health risks.

### Purchasing

#### Mitigation measures

Payment methods can be adjusted to incentivize health facilities and health workers to reduce their climate and environmental footprint (for example by changing service, prescription and dispensing practices, producing less waste, reducing energy consumption and preventing drug expiration).^6^^,^^7^ Under the concept of green purchasing,^10^ specific rules apply to the purchasing of drugs and commodities, as a lever for more climate-protective drug production. Closely related to purchasing, procurement rules and accreditation criteria could promote climate and environmental consciousness.

#### Adaptation measures

To make purchasing more strategic, that is, making the allocation of funds more efficient and needs-oriented, climate-change related health conditions and health services to address climate shocks and stress should be part of the benefits – instead of excluding them, as some insurance schemes do. Benefits will need to be flexible and quickly adaptable and in line with equity considerations to address the needs of different, vulnerable population groups.^6^^,^^7^ Purchasing agencies such as health ministries or national health insurance schemes should give more attention and funding to health prevention and promotion activities to prepare against heat-waves, flooding, vector-borne diseases and other climate-sensitive health risks, and adequately remunerate health providers who conduct these activities. Likewise, allocation decisions related to capital investments to make hospitals climate resilient need to be incentivized, which may be particularly important for Small Island Developing States.

Adjusting cost-sharing policies to support population groups that are vulnerable to climate shocks and stress will also be important, as well as securing the geographical portability of their benefits in times of climate change-induced migration and displacement.^11^ Likewise, the provision of emergency cash transfers could also be integrated into health benefits.

Box 2 illustrates two emerging practices in health financing policy in low- and middle-income countries.

Box 2 **Emerging country practice in health financing policy**

While adaptation and mitigation measures through health financing arrangements are not yet widespread, several countries have taken steps and introduced such measures, although little is published. For instance, with support from the World Bank, the Egyptian government has adopted the concept of green health insurance. Under this strategy, people most affected by climate change may be eligible for free or subsidized health insurance, while service providers will be incentivized to reduce their environmental footprint (for example by improving energy efficiency and reducing waste). A green benefits package would include health services to address climate-sensitive health risks, as well as more use of telemedicine. The initiative also aims to move towards a paperless health insurance administration through digitalization of all key administrative processes.^7^ In Ethiopia, the draft National Health Adaptation Plan includes initiatives in revenue raising (the use of green bonds to fund health sector projects with environmental benefits, such as renewable energy or access to clean water) as well as green purchasing, whereby the health sector is incentivized to buy products and services with reduced climate impact.^12^

Cutting across all health financing functions, public financial management arrangements will need to be adapted to allow flexible use of funds, including reallocation in case of climate emergencies. Another important cross-cutting mitigation measure is to move away from paper-based processes through digitalization of all administrative processes within the health financing functions (public financial management, enrolment, empanelment, targeting, claims management, payment, performance monitoring, reporting, etc.). However, the huge energy needs of digitalization are a downside to be considered. 

## Way forward

Given the wide variations globally in health financing, health sector carbon emissions and unmet health needs, a fair distribution of the remaining carbon budget for health gains will be critical.^13^ Countries that currently have high health sector carbon emissions will need to urgently decarbonize without sacrificing health system performance; while countries that currently still have low health sector carbon emissions will need to focus on increasing health system performance without increasing emissions, to the extent possible.^2^ Since many low-income countries are strongly challenged by climate shocks and stress, adaptation measures to maintain health are of utmost importance.

While health financing and climate financing are not under the same national ministries or global financing facilities, they can be of benefit to each other. As a first step, co-benefits of investments in health for climate mitigation and adaptation can be mapped and assessed, as well as the co-benefits of investments in climate mitigation in other sectors for health. In a more advanced scenario, financing in one sector can be actively leveraged to benefit the other sector, ideally moving towards a whole-of-government approach. More financial resources will inevitably be required for the health sector to protect human health from climate change, for instance to establish or expand surveillance and control programmes for infectious diseases; to recruit and retain health workers; and to retrofit health facilities to withstand more extreme weather events and long-term climate change effects.^2^ More documentation and dissemination of country examples and best practices from countries of all income levels are needed to broaden the knowledge about appropriate health financing options. 

Some key policy and research questions include: (i) which strategies seem most feasible and effective in terms of mitigation and adaptation? What could countries do? What should countries not do? (ii) which objectives are more easily achievable in the short term?; (iii) what is the role of regulation versus incentives? Which type of incentives and/or disincentives are most effective? (iv) how to ensure that the digital transformation of health financing systems is pursued in a climate-friendly (low carbon) way? (v) how to measure the contribution of health financing to climate change mitigation and adaptation?

Governments and health ministries, parliamentarians, purchasing agencies, researchers, the private sector, civil society actors and health workers can all play their part to ensure that the role of health financing policy for mitigation and adaptation receives more attention on the policy and research agenda. The objective is to exploit the potential of health financing policy to contribute to addressing climate change. By setting appropriate incentives and disincentives, health financing policy instruments could prevent or reduce negative effects on the climate and environment while optimizing positive contributions to building climate-resilient health systems.
